# Prevalence and Risk Associated With Bladder Injuries During Caesarean Section in a Secondary Care Hospital

**DOI:** 10.7759/cureus.59249

**Published:** 2024-04-29

**Authors:** Jaysingh Majhi, Brajendra N Mishra, Madan M Majhi

**Affiliations:** 1 Obstetrics and Gynaecology, Tata Steel Hospital, Joda, IND; 2 Surgery, Tata Steel Hospital, Noamundi, IND; 3 Community Medicine, Srirama Chandra Bhanja (SCB) Medical College, Cuttack, IND

**Keywords:** risk factor for bladder injury, complication of cesarean section, pregnant women, cesarean section, bladder injury

## Abstract

Introduction

Bladder injury during caesarean section (CS) is not uncommon. Various factors increase the risk of bladder injury during CS, including prolonged labor with bladder distension, pregnancy with a scarred uterus, suspected intra-abdominal adhesions, distorted local anatomy, cesarean hysterectomy, and an increasing number of previous CS. Vigilant preoperative assessment and surgical precision are essential to mitigate these risks and ensure optimal outcomes for mother and child.

Objectives

To find out the prevalence and risk factors associated with bladder injuries during caesarean section.

Methodology

Hospital-based retrospective record review of 3600 pregnant women who had undergone cesarean section during the period January 2015 to December 2023 were included in the study. Data was analyzed using SPSS software, version 22 (trial version) (IBM Corp., Armonk, NY). The Chi-square test and Fisher’s exact test were used. Ethical clearance was obtained from the Institutional Ethics Committee at Tata Main Hospital Noamundi (approval number NI/CMO/26/24).

Result

Bladder injury prevalence was reported to be 1.1%. Bladder injuries were significantly (p<0.0001) more among the CS cases with underlined complications as compared to CS cases without any underlined complications. Repeat CSs were found to have a significantly (p<0.001) higher proportion of bladder injuries compared to primary CS.

Conclusion

Bladder injuries during cesarean section are a significant concern. The risk factors identified, such as the number of previous cesarean sections and complications during pregnancy, highlight the importance of careful preoperative assessment and surgical precision to prevent such injuries.

## Introduction

Bladder injury during caesarean births, with primary rates at 0.2% and repeat at 0.6%, is rare but significant [1]. The American Academy of Family Physicians (AAFP) and the American College of Obstetricians and Gynecologists (ACOG) jointly assert that surgical delivery, including caesarean section (CS), is within the realm of family medicine. AAFP's training programs for residents and fellows encompass core obstetric skills, including CS, aiming to equip family physicians to perform CS independently [2].

The prevalence of morbidly adherent placenta in subsequent pregnancies is rising due to the increasing use of cesarean sections [2,3]. Morbid adherent placenta, comprising accreta, increta, and percreta, is associated with a higher incidence of bladder involvement during pregnancy, leading to more frequent bladder injuries. Various factors increase the risk of bladder injury during CS, including prolonged labor with bladder distension, pregnancy with a scarred uterus, suspected intra-abdominal adhesions, distorted local anatomy, cesarean hysterectomy, and an increasing number of previous CS [4]. Emergency CS and CS performed on laboring women also carry higher risks of bladder injury compared to elective CS and non-laboring women, respectively. Women with more than three previous CS are particularly prone to bladder injury, being five times more likely compared to those with only one previous CS [5].

Incidence outside India ranges from 0.104% to 0.28% [2,3]. Complications include prolonged surgery, urinary tract infections, and fistula formation [4-6]. Risk factors encompass emergency procedures, fetal positioning, labor onset, gestational age below 32 weeks, chorionic membrane rupture, prior caesarean history, and operator skill [7]. Vigilant preoperative assessment and surgical precision are essential to mitigate these risks and ensure optimal outcomes for mother and child [7]. Immediate recognition and management are crucial to mitigate complications. With this background, this study was conducted to find out the prevalence and risk factors associated with bladder injuries during caesarean section in a secondary care hospital.

## Materials and methods

This is a hospital-based cross-sectional study conducted over two months (February and March 2024). A record review of 3600 pregnant women who had undergone cesarean section at Tata Steel Mines Hospital Joda, Odisha, during the period January 2015 to December 2023 was done and was included in the study. A predesigned and pretested questionnaire was used for data collection.

Operational definition of bladder injury: During the record review, bladder injury is said to have occurred when the following points had been mentioned in the documents as per the guideline for Bladder Injury at Caesarean Birth Guideline adopted from National Health Service (NHS) Wales guidelines [8]: (i) Signs suggestive of a bladder injury i.e., visualization of urine in the operative field, visualization of transurethral Foley's catheter in the operative field, and hematuria; (ii) Confirmed bladder injury by instillation of diluted dye (methylene blue) through the transurethral catheter into the urinary bladder; (iii) Confirmed iatrogenic bladder injury of any grade from Grade1 to 5. Grade1 - contusion, intramural hematoma, or partial thickness laceration; Grade 2 - extraperitoneal bladder wall laceration <2 cm; Grade 3 - extraperitoneal >2 cm or intraperitoneal <2 cm laceration; Grade 4 - intraperitoneal bladder wall laceration >2 cm; Grade 5 - intra-or extraperitoneal bladder wall laceration involving the trigone or bladder neck. In absence of all of the above, if the document mentioned that there was bladder injury during CS that was also considered as bladder injury in this study.

Ethical clearance was obtained from the Institutional Ethics Committee, Tata Main Hospital Noamundi, vide Ref. No. NI/CMO/16/24 dated 05.02.2024. Data was analyzed using SPSS software, version 22 (trial version) (IBM Corp., Armonk, NY). The Chi-square test and Fisher’s exact test were used for inferential purposes. A p-value of <0.05 was considered for declaring a significant difference.

## Results

**Figure 1:**
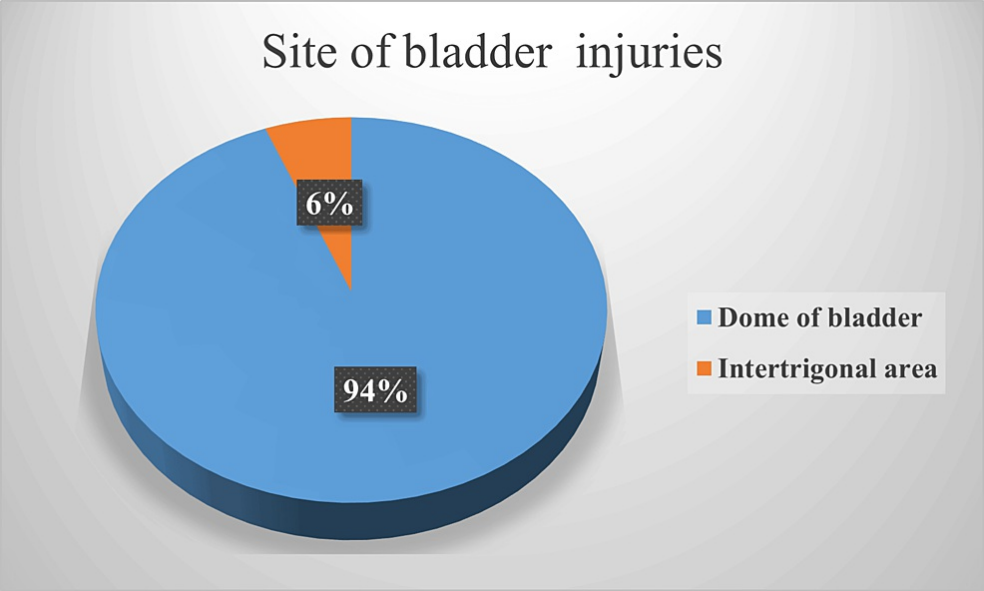
Site of bladder injury (N=40)

In total, 3600 pregnant women undergone CS during the period were analyzed and presented here. Among them, 40 (1.1%) cases of bladder injuries were reported. Figure 1 shows the most common site of bladder injury was the dome of the bladder (94%) (Figure 1).

General characteristics of patients with bladder injuries are given in Table 1.

**Table 1:** General characteristics of bladder injury patients(N=40) G: Gravidity; CS: Caesarian Section

Characteristics	Number	Percentage (%)
Age of pregnant mother		
18-25 years	10	25.0
25-35 years	12	30.0
>35 years	18	45.0
Gestational status		
G1	07	17.5
G2	10	25.0
G3	13	32.5
>G4	10	25.0
Stages of Labour		
In latent phase	05	12.5
In early active phase	10	25.0
In late active phase	25	62.5
Caesarean section without Complication (n=20)		
Primary CS	02	10.0
Repeat one CS	03	15.0
Repeat two CS	10	50.0
Repeat three CS	05	25.5
Caesarean section with Complication (n=20)		
Pregnancy with h/o Endometriosis	04	20.0
Post Myomectomy Pregnancy	06	30.0
Ruptured Uterus	10	50.0

Most (45.0%) of the bladder injury patients were in the age group of <35 years. Third gravida patients had most (32.5%) of the blade injuries. About 62% of injuries were reported among patients undergoing CS during the late active phase of labour. Among the total cases with bladder injury, 20 (50.0%) cases were reported in patients who had undergone CS without complication and another 50% in patients who had undergone CS for any underlying Complication. Risk factors for bladder injuries are given in Table 2. 

**Table 2:** Risk factor for bladder injuries among pregnant women (N=3600) CS: Caesarean section; Figure in bracket indicates percentage

Patient Profile		Patients	Blader Injury
CS without Complication	Primary CS	1650	2 (0.12)
	Repeat one CS	1590	3 (0.18)
	Repeat two CS	285	10 (3.5)
	Repeat three CS	25	5 (20.0)
CS with Complication	Pregnancy with h/o endometriosis	15	4 (26.0)
	Post myomectomy pregnancy	10	6 (60.0)
	Ruptured uterus	25	10 (40.0)

In all pregnant women under CS, 20% of cases of bladder injuries were reported in those with repeat three CS, whereas the injuries were only 3.5% in those with repeat two CS and negligible in primary CS and repeat one CS. The majority (60.0%) cases of bladder injuries were reported in Post Myomectomy Pregnancy patients undergoing CS. Bladder injuries reported were 40% and 26% among the patients with Ruptured Uterus and Pregnancy with h/o Endometriosis, respectively. Bladder injuries were significantly(p<0.0001) more among the CS cases with underlined complications as compared to CS cases without any underlined Complication (Table 3).

**Table 3:** Association of bladder injury in patients with caesarian section with or without Complication (N=3600) CS: Caesarean section; Figures in brackets indicate the percentage

Patient profile	n	Bladder Injury		p-value
		Yes	No	
CS without Complication	3500	20(0.56)	3530 (99.44)	0.0001
CS with Complication	50	20(40.0)	30 (60.0)	

Repeat CS was found to have a significantly (p<0.001) higher proportion of bladder injuries compared to primary CS (Table 4).

**Table 4:** Association of bladder injuries among CS cases with Complication (N=3550) CS: Cesarian section; Figures in bracket indicate the percentage

Patient Profile	n	Bladder Injury		p-value
		Yes	No	
Primary CS	1650	2(0.12)	1648(99.88)	<0.001
Repeat one CS	1590	3(0.18)	1587(99.82)	
Repeat two CS	285	10(3.50)	275(96.50)	
Repeat three CS	25	5(20.0)	20(80.00)	

Whereas the types of complications were not found to be significantly associated with bladder injury (Table 5).

**Table 5:** Association of bladder injuries among CS cases with Complication (N=50) Figures in brackets indicate the percentage

Patient Profile	n	Bladder Injury		p-value
		Yes	No	
Pregnancy with a past history of Endometriosis	15	4(26.66)	11(73.34)	0.24
Post Myomectomy Pregnancy	10	6(60.00)	4(40.00)	
Ruptured Uterus	25	10(40.00)	15(60.00)	

## Discussion

In the current study, 1.1% of bladder injury cases were found among all the patients who underwent CS, while Al-Shahrani M, in his study conducted in 2012, reported 0.22% of bladder injury among the participants [9]. A similar proportion of 0.2% bladder injury was reported by Ibrahim et al. in their study conducted from 1999-2015 among 41,69,681 CS deliveries [10]. An even lower proportion of bladder injury, i.e. 0.08%, has been reported by Safrai et al. in their study conducted between 2004-2018, including 17,794 CS deliveries [11]. Similar results have been reported by Salman et al. in their study conducted during 2007-2016 among 17.326 CS deliveries found that 0.07% had bladder injuries [12].

In this study, in 94% of cases, the site of bladder injury was the dome of the bladder (Figure 1). A similar finding was reported by Philip et al. [2]. Regarding the timing of bladder injuries, most bladder injuries (53%) are encountered while entering the peritoneal cavity due to extensive adhesions, unexpectedly high-situated bladder due to previous operations, and also due to distorted pelvic anatomy [13]. A few authors also reported that surgical adhesions are a risk factor for bladder injury [14,15]. Out of 13 urinary bladder injuries reported, four (30.7%) were detected in post-operative periods [16]. Along with bladder injuries, ureteric injuries may also be encountered. Gangai et al. reported an incidence of ureteric injury up to 0.2-1.0% during any abdominal or pelvic surgery [17].

In the current study, bladder injuries were found to be significantly more common among patients who had CS with complications. A similar result has been reported by Al-Shahrani M, who compared the bladder injury with estimated blood loss and found a higher proportion of participants with >1000 ml having bladder injury, which was found to be statistically significant [9]. A similar statistical association was found with low birth weight, which was another complication studied by Al-Shahrani M [9]. In our study, women having prior three CS were at a higher risk of bladder injury, which was found to be statistically significant similar to the findings of Al-Shahrani M [9].

The risk of bladder injury among complicated pregnancies was found to be significantly higher in our study, which is similar to the findings of Ibrahim et al., who reported Endometriosis and previous cesarean delivery as significant risk factors with Odd’s ratio of 2.04 and 4.37, respectively [10]. Similarly, Safrai et al. reported in their study that women who underwent emergency CS (Complicated CS) and a previous history of CS were at higher risk of Bladder injury, which was statistically significant (p<0.05) [11].

As this is a cross-sectional study, the causality of risk factors can not be ascertained. However, the strength of this study is that it delineated a few important factors, and the presence of those factors is associated with bladder injuries in a remote mining area of Odisha.

## Conclusions

Bladder injuries during caesarean section are a significant concern, with a prevalence of 1.1% in the current study. The risk factors identified, such as the number of previous cesarean sections and complications during pregnancy, highlight the importance of careful preoperative assessment and surgical precision to prevent such injuries. The findings underscore the need for increased awareness and vigilance among obstetric surgeons to minimize the occurrence of bladder injuries during caesarean deliveries.
